# MicroRNA-148a-3p is a candidate mediator of increased bone marrow adiposity and bone loss following spinal cord injury

**DOI:** 10.3389/fendo.2022.910934

**Published:** 2022-08-05

**Authors:** Samantha Lincoln, Leslie R. Morse, Karen Troy, Nicole Mattson, Nguyen Nguyen, Ricardo A. Battaglino

**Affiliations:** ^1^ Drexel University College of Medicine, Philadelphia, PA, United States; ^2^ Department of Rehabilitation Medicine, University of Minnesota School of Medicine, Minneapolis, MN, United States; ^3^ Department of Biomedical Engineering, Worcester Polytechnic Institute, Worcester, MA, United States

**Keywords:** spinal cord injury, osteoporosis, microRNA, biomarker, rehabilitation medicine

## Abstract

Spinal cord injury is often followed by osteoporosis characterized by rapid and severe bone loss. This leads to an increased risk of osteoporotic fracture in people with spinal cord injury, resulting in increased healthcare costs, morbidity, and mortality. Though it is common, the mechanisms underlying this osteoporosis are not completely understood and treatment options are limited. No biomarkers have been identified for predicting fracture risk. In this study, we sought to investigate microRNA mediated mechanisms relating to osteoporosis following spinal cord injury. We studied subjects with acute SCI (n=12), chronic SCI (n=18), and controls with no SCI (n=23). Plasma samples from all subjects underwent transcriptomic analysis to quantify microRNA expression, after which miR-148a-3p was selected for further study. We performed CT scans of the knee on all subjects with SCI and analyzed these scans to quantify bone marrow adipose tissue volume. MiR-148a-3p was upregulated in subjects with acute SCI vs chronic SCI, as well as in acute SCI vs no SCI. Subjects with chronic SCI had greater levels of marrow adiposity in the distal femoral diaphysis compared to subjects with acute SCI. MiR-148a-3p levels were negatively associated with distal femoral diaphysis marrow adiposity. A multivariable model showed that miR-148a-3p and BMI explained 24% of variation in marrow adiposity. A literature search revealed that miR-148a-3p has multiple bone and fat metabolism related targets. Our findings suggest that miR-148a-3p is a mediator of osteoporosis following spinal cord injury and a potential future therapeutic target.

## Introduction

Spinal cord injury (SCI) is often followed by osteoporosis characterized by rapid and severe bone loss. Osteoporosis occurs when the highly regulated balance between bone resorption and bone formation becomes uncoupled, leading to declines in bone mineral density (1, 2). This bone loss can lead to an increased risk of fractures in people with SCI, resulting in longer hospital stays, as well as increased morbidity (3–5). Of note, there are currently no biomarkers for bone loss or fracture risk, representing a difficulty in identifying people with SCI who are at most risk of developing these complications. Additionally, the underlying mechanisms mediating SCI induced osteoporosis are not fully understood and treatment options remain limited.

MicroRNAs (miRNAs) are 19-24 nucleotide noncoding RNAs that were first discovered in C. elegans, and have since been identified in many species, including humans (6, 7). MiRNAs act as suppressors of their target genes by binding to their mRNAs, leading to either translational repression or complete degradation of the mRNA transcript (8). They are thought to regulate at least one-third of the human genome and are involved in many physiological processes (9). Differential expression of microRNAs cellularly and in the circulation has been implicated in several disease processes, demonstrating their role in pathophysiology and their potential as biomarkers for disease (10–12). MiR-148a-3p, in particular, has been shown to be elevated in the plasma and bone marrow of individuals with osteoporosis (13, 14), in the plasma of individuals with Type 1 Diabetes related bone loss (15), and in the adipose tissue of individuals with obesity (16).

To date, there is limited information on the role miRNAs as regulators of osteoporosis after SCI. Therefore, the goal of this study was to assess the association between miR-148a-3p and bone parameters in individuals with SCI.

## Materials and methods

### Subjects

We studied a convenience sample of participants with SCI and uninjured controls who participated in one of two ongoing clinical trials. From the first clinical trial, participants with SCI were included if they were 18 years of age or older. The injury duration had to be equal or longer than 3 years. The primary means of mobility was wheelchair. The SCI level ranged from C7 to T12, height of 155-191 centimeters, weight of less than 113 kg, spasticity in both lower extremities of less than 3 on the Modified Ashworth Scale (MAS), and the individuals had to have sufficient upper body strength to complete sit to sit transfers. Exclusion criteria included: enrollment in another clinical trial, pregnancy, orthostatic hypotension with fall in blood pressure >30 mmHg when upright, an active pressure ulcer (grade 2 or higher) an unhealed limb or pelvic bone fracture, history of other neurological disease (e.g. stroke, peripheral neuropathy, myopathy), active treatment for epilepsy or thyroid disorders, active use of medications that might affect bone health (bisphosphonates, androgenic steroids, estrogenic steroids, lithium, glucocorticoids). Participants without SCI were: 18 years of age or older. These control subjects were matched to the first 12 enrolled participants with SCI by age, gender, and ethnicity. Pregnant women without SCI were excluded. From the second clinical trial we included participants with acute SCI (less than 3 months after injury) who were 18-60 years of age and also used a wheelchair as their primary mode of mobility. From this trial, participants were excluded if they were enrolled in another clinical trial or had contraindications to simvastatin. Additional exclusion criteria can be found in (17) and included metabolic bone disease, untreated thyroid disease, history of bilateral oophorectomy, active use of medications potentially affecting bone health (bisphosphonates, androgenic steroids, estrogenic steroids, anti-epileptics and lithium, glucocorticoids), and those who received inhaled glucocorticoids in the past year, pregnant or lactating women and women of childbearing potential who were unwilling or unable to use a reliable form of contraception.

Genome wide screening was completed on n=63 participants (n=23 no SCI, n=14 acute SCI, n=26 chronic SCI). We excluded 8 chronic SCI and 1 acute SCI without available CT data, and 1 acute SCI with missing data. The final cohort consisted of 53 men and women, with and without SCI (n=23 no SCI, n=12 acute SCI, n=18 chronic SCI) who completed baseline testing between 08/04/2017 and 11/05/2019. The Institutional Review Board approved all protocols prior to initiation of the study, and all participants gave their written informed consent to participate.

### Variable definition

At the time of enrollment, the participants completed a questionnaire, from which we obtained the information regarding SCI, medical history, health habits, and medication use. Injury level was considered dichotomously: paraplegia vs tetraplegia. Age, age at injury, and duration of injury, on the other hand, were considered as continuous variables.

### Motor score

Motor level and completeness of injury were confirmed by a physical exam by the study physician according to the American Spinal Injury Association Impairment Scale (AIS) as described (18, 19). Participants were classified as AIS A (sensory and motor complete, no sensory or motor function below the neurological level of injury), AIS B (motor complete, preservation of sensory but no motor function below the neurological level of injury) or AIS C (motor incomplete, sensory and motor function preserved below the neurological level, and more than half the key muscles below the neurological level are not strong enough to overcome gravity).

### Volumetric quantitative computed tomography analysis

We scanned distal femur and proximal tibia of individuals with acute SCI and with chronic SCI using a 16-slice multi-detector row CT scanner (GE Lightspeed, 120 kVp, 50 mAs, pixel resolution 0.652 to 0.977 mm, slice thickness 0.625 to 1.250 mm). Each scan included a calibration phantom (MindWays, Austin, TX, USA) in the imaging field to convert CT Hounsfield units (HU) to bone-equivalent density (rHA, g/cm3). Reconstructed images comprised a 18- to 25-cm region surrounding the knee joint. After each bone was aligned anatomically, a fixed density threshold of 0.15 g/cm3 was used to identify the periosteal surface of bone. In some images, manual correction was required to fill in missing low-density surface voxels; all images were processed by a single investigator (NM).

Scans were divided into cortical and trabecular regions by eroding the outermost portion of the bone, the cortical region, by the number of pixels determined by dividing 3.5mm by the pixel size. Within the inner trabecular region of the bone, MATLAB (Mathworks, MA, US) was used to isolate the regions with density in Hounsfield units (HU) by performing a thresholding between -205 and -50 and calculate the volume of MAT at the epiphysis, metaphysis, and diaphysis. The epiphysis, metaphysis, and diaphysis were determined by using 10%, 20%, and 30% of the total length from the distal femur or proximal tibia.

### Biochemical analyses

Blood samples were drawn into an EDTA tube and immediately delivered to the core blood research laboratory at our facility for processing. Samples were then: 1) centrifuged for 15 min at 2600 rpm (1459 x g) at 40C and 2) stored at -80^0^C until analysis. All microRNA analyses were performed by LC Sciences at their facilities in Houston, TX, as previously described (17, 20). Briefly, a comprehensive miRNA/small RNA sequencing service was performed, which included: sample QC, library preparation, and sequencing (50 base pair sequencing, on average a minimum of 7-10 million reads per sample). Raw reads were subjected to an in-house program, ACGT101-miR (LC Sciences, Houston, Texas, USA) to remove adapter dimers, junk, low complexity, common RNA families (rRNA, tRNA, snRNA, snoRNA) and repeats. Subsequently, unique sequences with length in 18~26 nucleotide were mapped to specific species precursors in miRBase 21.0 by BLAST search to identify known miRNAs and novel 3p- and 5p- derived miRNAs. Length variation at both 3’ and 5’ ends and one mismatch inside of the sequence were allowed in the alignment. The unique sequences mapping to specific species mature miRNAs in hairpin arms were identified as known miRNAs. The unique sequences mapping to the other arm of known specific species precursor hairpin opposite to the annotated mature miRNA-containing arm were novel 5p- or 3p- derived miRNA candidates. The remaining sequences were mapped to other selected species precursors (with the exclusion of specific species) in miRBase 21.0 by BLAST search, and the mapped pre-miRNAs were further BLASTed against the specific species genomes to determine their genomic locations. The unmapped sequences were BLASTed against the specific genomes, and the hairpin RNA structures containing sequences were predicated from the flank 80 nt sequences using RNAfold software (http://rna.tbi.univie.ac.at/cgi-bin/RNAWebSuite/RNAfold.cgi). The criteria for secondary structure prediction were: (1) number of nucleotides in one bulge in stem (≤12), (2) number of base pairs in the stem region of the predicted hairpin (≥16), (3) cutoff of free energy (kCal/mol ≤-15), (4) length of hairpin (up and down stems + terminal loop ≥50), (5) length of hairpin loop (≤20), (6) number of nucleotides in one bulge in mature region (≤8), (7) number of biased errors in one bulge in mature region (≤4), (8) number of biased bulges in mature region (≤2), (9) number of errors in mature region (≤7), (10) number of base pairs in the mature region of the predicted hairpin (≥12), and (11) percent of mature in stem (≥80).

Normalization of sequence counts in each sample (or data set) was achieved by dividing the counts by a library size parameter of the corresponding sample. The library size parameter is a median value of the ratio between the counts a specific sample and a pseudo-reference sample. A count number in the pseudo-reference sample is the count geometric mean across all samples.

$s_{j}=\underset{i}{median}\left(\frac{c_{ij}}{{\left(\prod_{k=1}^{m}c_{ik}\right)}^{1/m}}\right)$ ,where Sj is the library size parameter; cij is the count number of sequence i of sample j; m is the total number of samples involved.

### Target prediction analysis

Target genes possibly regulated by miRNAs of interest were detected using the consensus of three publicly available miRNA target databases: TargetScan (http://www.targetscan.org), MicroCosm (http://www.ebi.ac.uk/enright-srv/microcosm), and miRTarBase (http://mirtarbase.mbc.nctu.edu.tw). The predicted target false-positive rate was reduced significantly by applying a cut-off of –0.4 in the context scores for TargetScan results. Enrichment and pathway analysis of predicted miRNA gene targets was performed using MetaCoreTM process networks, pathway maps and GO molecular functions/processes. Interactions between the miRNA and their target gene networks were pictured using CyTargetLinker v3.0.1, an software package for Cytoscape v.4.0.43. Circos v0.67 was used to show interaction between miRNA and target genes in a circular layout, facilitating the visualization of the position of the miRNA and target genes. Targets with strong experimental evidence for hsa-mir-148a-3p were retrieved, following which, an extensive literature search of the PubMed database was performed to further confirm bone and fat metabolism associated with the validated targets.

### Statistical analysis

Differential expression of miRNA was determined based on injury type (No SCI n=23, acute SCI n=12, and chronic SCI n=18). For each comparison, normalized deep-sequencing counts were analyzed by selectively using Fisher exact test, Chi-squared 2X2 test, Chi-squared nXn test, Student t test, or ANOVA based on the experiments design. The significance threshold was set to be 0.01 and 0.05 in each test. All subsequent analyses were performed using SAS 9.4 (SAS Institute, Inc., Cary, NC). T-tests or χ^2^ tests were used to compare subject characteristics as appropriate. General linear models (PROC GLM) were applied to assess associations between biomarker levels (miR-148a-3p), marrow adiposity, and total bone mineral content. Distal femoral marrow adiposity was selected for multivariable model building based on univariate findings. Factors with a *p* value of <0.10 in the univariate models were included in the multivariable models. Factors with a *p* value of <0.05 were considered statistically significant.

## Results

### Subject characteristics

Subject characteristics are presented in **Tables 1A**, **B**. Participants with No SCI were 37.1 ± 11.1 years of age (range 24.2-57.9 years) and 57% male. Participants with acute SCI were 36.9 ± 9.5 years of age (range 26.6-54.7 years), 83% male, and 2.7 ± 0.6 months post injury (range 2.0-4.3 months). Participants with chronic SCI were 34.4 ± 9.5 years of age (range 18.6-51.0 years), 78% male, and 10.1 ± 7.2 years post injury (range 3.1–23.4 years). All participants with SCI used a wheelchair as their primary mode of mobility. Those with acute SCI were more likely to be tetraplegic than those with chronic SCI (p=0.01, **Table 1B**).

**Table 1A:** Characteristics of cohort.

	No SCI (n=23)	Acute SCI (n=12)	Chronic SCI (n=18)	Total (n=53)	p
Age (years) [Mean ± SD]	37.08 ± 11.07	36.88 ± 9.45	34.43 ± 9.51	36.14 ± 10.09	0.68
Years post-injury [Mean ± SD]	N/A	0.23 ± 0.05	10.08 ± 7.19	6.14 ± 7.37	<0.0001^*^
Age at injury (years) [Mean ± SD]	N/A	36.67 ± 9.46	24.36 ± 7.77	29.29 ± 10.34	0.0006^*^
Males, n (%)	13 (56.5)	10 (83.3)	14 (77.8)	24 (80.0)	0.17
ASIA Classification	N/A				1.0^*^
A, n(%)		9 (75.0)	13 (72.2)	22 (73.3)	
B, n(%)		2 (16.7)	3 (16.7)	5 (16.7)	
C, n(%)		1 (8.3)	2 (11.1)	3 (10.0)	
Tetraplegic, n(%)	N/A	7 (58.3)	2 (11.1)	9 (30.0)	0.01^*^
Mir148a-3p (normalized deep sequencing count) [Mean ± SD]	9731.65 ± 4406.23	13241.08 ± 2927.81	9407.89 ± 2463.35	10416.28 ± 3796.29	0.01^^^
Mir148a-5p (normalized deep sequencing count) [Mean ± SD]	88.30 ± 48.13	130.16 ± 64.73	92.11 ± 27.17	99.07 ± 48.98	0.03^^^^
Body Mass Index (BMI) (kg/m^2^) [Mean ± SD]	24.22 ± 3.21^a^	23.51 ± 4.30	23.28 ± 4.66	23.72 ± 3.97^a1^	0.75
Apparent Bone Mineral Density (g/cm^2^) [Mean ± SD]	N/A				
Left femoral neck		0.96 ± 0.14	0.81 ± 0.14	0.87 ± 0.15	0.007^*^
Left total hip		0.96 ± 0.16	0.75 ± 0.10	0.83 ± 0.16	0.0002^*^
Right femoral neck		0.98 ± 0.13	0.77 ± 0.10	0.85 ± 0.15	<0.0001^*^
Right total hip		0.96 ± 0.15	0.72 ± 0.09	0.82 ± 0.17	<0.0001^*^
Diaphysis marrow adiposity volume (cm^3^) [Mean ± SD]	N/A	3.45 ± 1.77	7.80 ± 4.08	6.06 ± 3.95	0.0005^*^
Diaphysis trabecular bone mineral content (g) [Mean ± SD]	N/A	1.19 ± 0.92	-0.52 ± 0.63	0.15 ± 1.13	<0.0001^*^
Epiphysis trabecular bone mineral content (g) [Mean ± SD]	N/A	10.79 ± 3.48	3.22 ± 2.10	6.25 ± 4.62	<0.0001^*^
Metaphysis trabecular bone mineral content (g) [Mean ± SD]	N/A	3.24 ± 1.79	-0.24 ± 1.26	1.14 ± 2.27	<0.0001^*^
Total trabecular bone mineral content (g) [Mean ± SD]	N/A	15.22 ± 5.95	2.44 ± 3.47	7.56 ± 7.81	<0.0001*

*among SCI only.

^^^p=0.02 (no SCI vs acute SCI), p=0.01 (chronic SCI vs acute SCI), p=0.95 (no SCI vs chronic SCI).

^^^^p=0.04 (no SCI vs acute SCI), p=0.08 (chronic SCI vs acute SCI), p=0.96 (no SCI vs chronic SCI).

a available out of n=21, ^a1^available out of n=51. N/A, not applicable.

**Table 1B:** Characteristics of SCI cohort.

	Acute SCI (n=12)	Chronic SCI (n=18)	Total (n=30)	p
Age (years) [Mean ± SD]	36.88 ± 9.45	34.43 ± 9.51	35.41 ± 9.40	0.49
Years post-injury [Mean ± SD]	0.23 ± 0.05	10.08 ± 7.19	6.14 ± 7.37	<0.0001
Age at injury (years) [Mean ± SD]	36.67 ± 9.46	24.36 ± 7.77	29.29 ± 10.34	0.0006
Males, n (%)	10 (83.3)	14 (77.8)	24 (80.0)	1.0
ASIA Classification				1.0
A, n(%)	9 (75.0)	13 (72.2)	22 (73.3)	
B, n(%)	2 (16.7)	3 (16.7)	5 (16.7)	
C, n(%)	1 (8.3)	2 (11.1)	3 (10.0)	
Tetraplegic, n(%)	7 (58.3)	2 (11.1)	9 (30.0)	0.01
Mir148a-3p (normalized deep sequencing count) [Mean ± SD]	13241.08 ± 2927.81	9407.89 ± 2463.35	10941.17 ± 3233.67	0.0006
Mir148a-5p (normalized deep sequencing count) [Mean ± SD]	130.16 ± 64.73	92.11 ± 27.17	107.33 ± 48.80	0.07
BMI (kg/m^2^) [Mean ± SD]	23.51 ± 4.30	23.28 ± 4.66	23.38 ± 4.45	0.89
Bone Density (g/cm^2^) [Mean ± SD]				
Left femoral neck	0.96 ± 0.14	0.81 ± 0.14	0.87 ± 0.15	0.007
Left total hip	0.96 ± 0.16	0.75 ± 0.10	0.83 ± 0.16	0.0002
Right femoral neck	0.98 ± 0.13	0.77 ± 0.10	0.85 ± 0.15	<0.0001
Right total hip	0.96 ± 0.15	0.72 ± 0.09	0.82 ± 0.17	<0.0001
Diaphysis marrow adiposity volume (cm^3^) [Mean ± SD]	3.45 ± 1.77	7.80 ± 4.08	6.06 ± 3.95	0.0005
Diaphysis trabecular bone mineral content (g) [Mean ± SD]	1.19 ± 0.92	-0.52 ± 0.63	0.15 ± 1.13	<0.0001
Epiphysis trabecular bone mineral content (g) [Mean ± SD]	10.79 ± 3.48	3.22 ± 2.10	6.25 ± 4.62	<0.0001
Metaphysis trabecular bone mineral content (g) [Mean ± SD]	3.24 ± 1.79	-0.24 ± 1.26	1.14 ± 2.27	<0.0001
Total trabecular bone mineral content (g) [Mean ± SD]	15.22 ± 5.95	2.44 ± 3.47	7.56 ± 7.81	<0.0001

### Differential expression of miR-148a-3p levels

**Figure 1:**
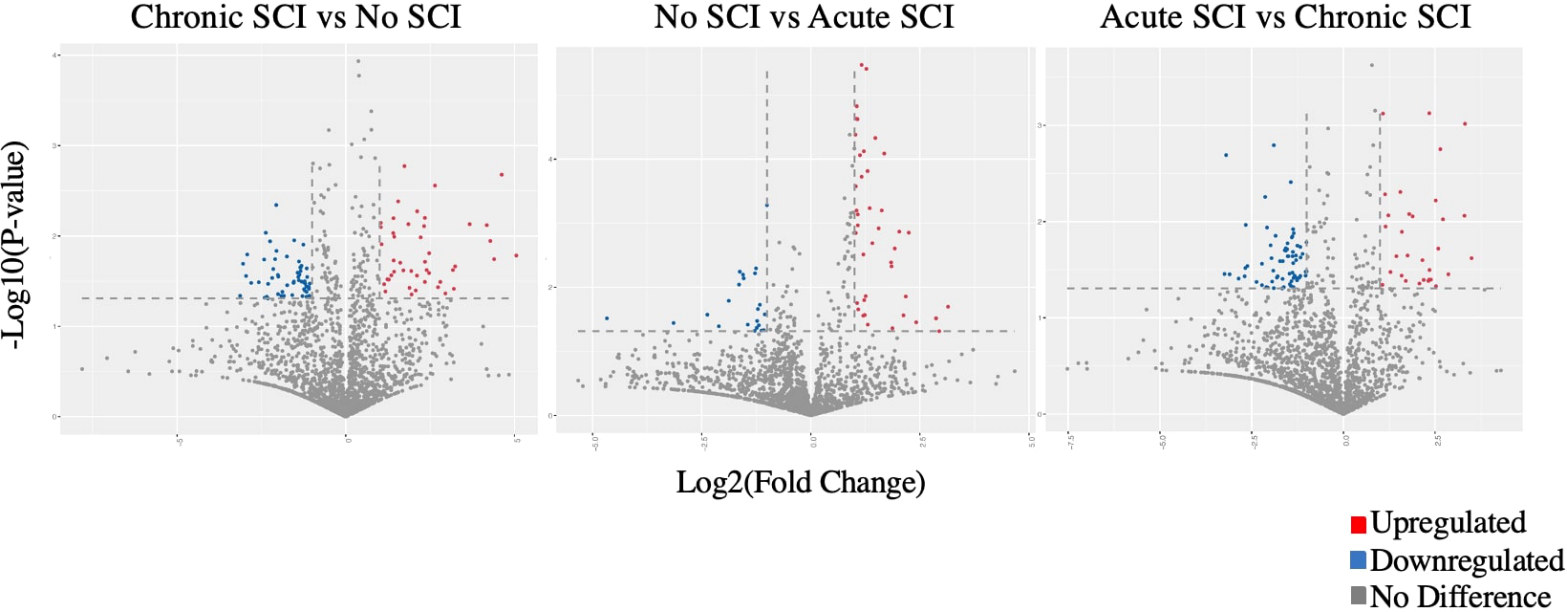
Volcano plots comparing Log2 (fold change) of miRNA expression values for participants who have a chronic SCI versus no SCI, no SCI vs acute SCI, and acute SCI vs chronic SCI. Significantly upregulated genes shown in red, significantly downregulated genes shown in blue (p<0.05). Some dots may represent more than one data point.

**Figure 2:**
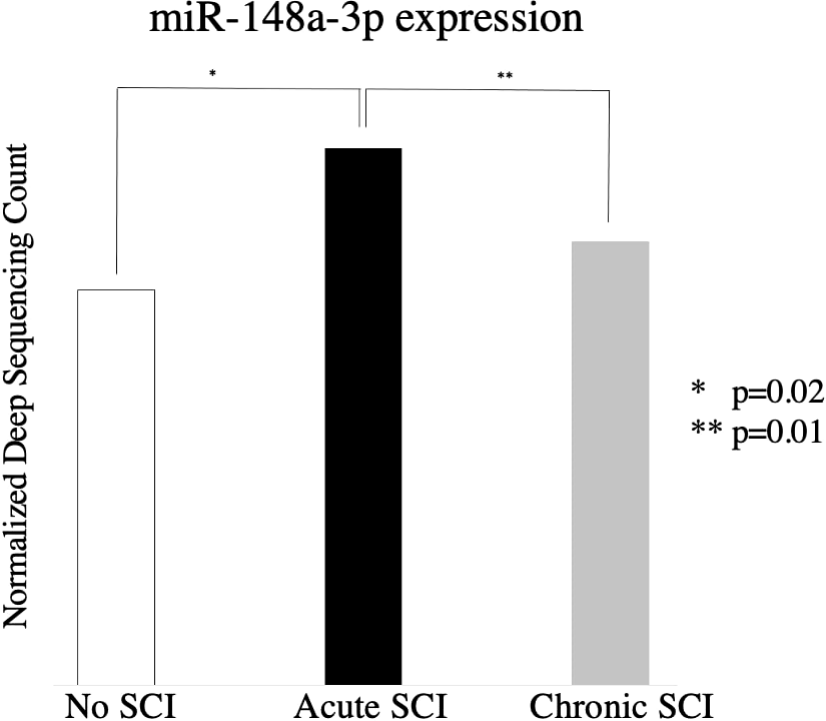
Relative expression of miR-148a-3p in subjects with no SCI, subjects with acute SCI, and subjects with chronic SCI.

706 miRNAs were significantly (p < 0.05) up- or down-regulated in acute SCI (255) or chronic SCI (451) versus no SCI. Additionally, 380 miRNAs were significantly (p < 0.05) up- or down-regulated in acute SCI versus chronic SCI (**Figure 1**). We selected miR-148a-3p for further analysis based on its upregulation in both acute SCI versus no SCI (13241.08 ± 2927.81 vs 9731.65 ± 4406.23, p=0.02) and acute SCI vs chronic SCI (13241.08 ± 2927.81 vs 9407.89 ± 2463.3, p=0.01) (**Figure 2**).

### Univariate factors associated with marrow adiposity at the distal femoral diaphysis

Among participants with SCI, injury duration (categorically) is significantly associated with marrow adiposity volume at the distal femoral diaphysis (**Table 2**). Subjects with chronic SCI had greater amounts of marrow adiposity at the distal femoral diaphysis than did subjects with acute SCI (7.80cm^3^ vs 3.45cm^3^, p=0.002). MiR-148a-3p and miR-148a-5p levels were negatively associated with marrow adiposity at the distal femoral diaphysis (p=0.07 and p=0.48, respectively).

**Table 2:** Univariate factors associated with marrow adiposity at the distal femoral diaphysis among acute and chronic SCI, n=30.

Continuous Variable	β ± SE	p
Age (cm^3^/year)	-0.02 ± 0.07	0.72
Injury duration (cm^3^/year)	0.01 ± 0.10	0.90
BMI (cm^3^/kg/m^2^)	0.27 ± 0.15	0.10
Mir148a-3p (cm^3^/normalized deep sequencing count)	-0.0004 ± 0.0002	0.07
Mir148a-5p (cm^3^/normalized deep sequencing count)	-0.01 ± 0.01	0.48
Categorical Variable	Mean Marrow Adiposity Volume (cm^3^) ± SE	
**Sex**		0.85
Male	5.99 ± 3.59	
Female	6.34 ± 5.59	
**Injury Duration**		0.002
Acute Injury (less than 1 year after injury)	3.45 ± 1.77	
Chronic (3 or more years after injury)	7.80 ± 4.08	

### Multivariable factors associated with marrow adiposity at the distal femoral diaphysis

In participants with SCI, BMI was positively associated with marrow adiposity at the distal femoral diaphysis (**Table 3**, p=0.04) while miR-148a-3p was negatively associated with marrow adiposity at the distal femoral diaphysis (p=0.03). This model explains 24% of the variation in marrow adiposity volume at the distal femoral diaphysis (p=0.03, R^2 =^ 0.24).

**Table 3:** Multivariable factors associated with marrow adiposity at the distal femoral diaphysis among acute and chronic SCI, n=30.

	Model p=0.03, R^2^ = 0.24	
	**β ± SE**	**p**
BMI (cm^3^/kg/m^2^)	0.32 ± 0.15	0.04
Mir148a-3p (cm^3^/normalized deep sequencing count)	-0.0004 ± 0.0002	0.03

### Validated targets of miR-148a-3p

Targets with strong experimental evidence for miR-148a-3p were retrieved from miRTarBase, following which, we performed an extended literature search of the PubMed database and further confirmed those bone and fat metabolism-associated targets, presented in **Table 4**. Promoters of adipogenesis and inhibitors of osteoblastogenesis DNMT1, IGF1, KDM6B, WNT1 and WNT10B are all validated targets of miR-148a-3p. miR-148a-3p also targets V-maf musculoaponeurotic fibrosarcoma oncogene homolog B (MAFB), a negative regulator of osteoclastogenesis (26)

**Table 4:** Bone and fat metabolism related validated targets of miR-148-3p.

Target	Model	microRNA effect	References
DNMT1	HeLA S3 cells	Downregulation of DNMT	Sharma et al. (21)
IGF1	Rat BMSCs	Reduced bone callus strength	Liu et al. (22)
MAFB	CD14+ Peripheral mononuclear blood cells	Promotion of osteoclastogenesis, increased bone resorption	Cheng et al. (23)
KDM6B	ST2 cells	Inhibition of osteoblastogenesis, promotion of adipogenesis	Tian et al. (24)
WNT1	Human mesenchymal stem cells-Ad	Inhibition of osteoblastogenesis, promotion of adipogenesis	Shi et al. (16)
WNT10B	3T3-L1 cells	Inhibition of osteoblastogenesis, promotion of adipogenesis	Cho et al. (25)

## Discussion

We evaluated the differential expression of miRNAs in subjects with chronic SCI, acute SCI, and no SCI and sought to investigate the association of these miRNAs with SCI mediated osteoporosis and related bone metabolism pathways. We identified an increase in the expression of miR-148-3p during the acute phase of spinal cord injury. We found that miR-148a-3p was negatively associated with marrow adiposity at the distal femoral diaphysis; and the negative association was significant after adjusting for BMI. Our studies are consistent with previous studies showing an association between osteoporosis and miR-148-3p (13).

MiR-148a-3p has several validated targets involved in the regulation and fate determination of bone marrow mesenchymal stem cells. Bone marrow mesenchymal stem are able to differentiate into multiple different kinds of cells, including osteoblasts and adipocytes (25). This balance is crucial in the maintenance of bone density and becomes dysregulated in several bone-related pathologies, including osteoporosis (24). miR-148a-3p has several targets that are involved in the regulation of this pathway, including WNT1 (16) and WNT10B (22). Suppression of these genes leads to a decrease in the Wnt/Frizzled signaling pathway, ultimately resulting in decreased osteoblastogenesis and increased adipogenesis of MSCs. miR-148a-3p also targets KDM6B (21), a histone demethylase involved in osteoblastic differentiation, as well as DNMT1 (23), a DNA methyltransferase that regulates adipogenesis. In addition, miR-148a-3p has also been shown to target IGF1, which has been shown to play a role in bone fracture healing (27).

MiR-148a-3p also targets MAFB, a transcription factor that inhibits osteoclastogenesis. Increased levels of miR-148a-3p lead to an osteoclastogenic environment *in vitro*, resulting in increased osteoclastic activity and potentially higher rates of bone resorption, further contributing to osteoporotic changes (26).

**Figure 3:**
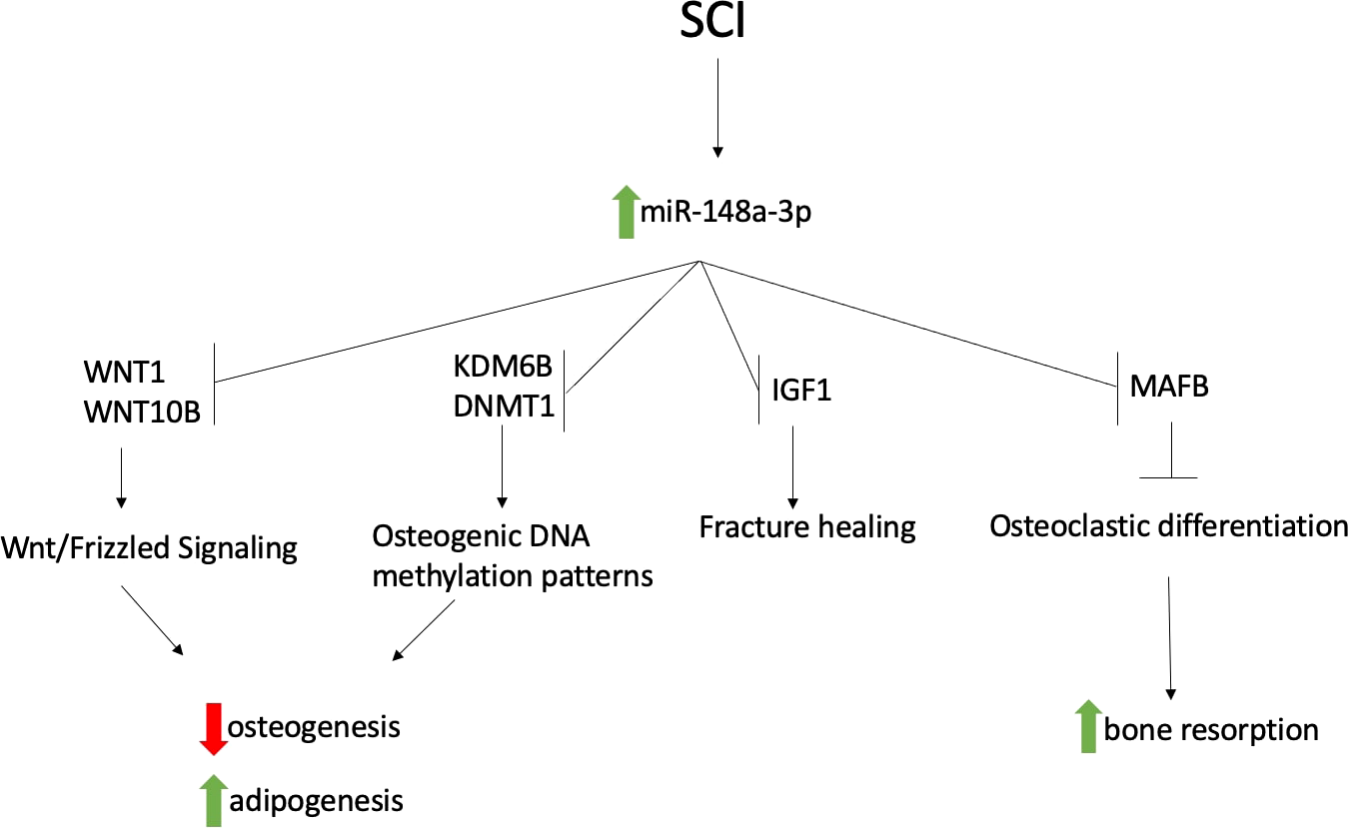
Proposed schematic of how increased miR-148a-3p during acute SCI contributes to osteoporosis following SCI.

This study showed a significant increase in miR-148a-3p during the acute phase of SCI compared to chronic SCI. Further analysis showed that this miRNA has multivariable and univariate associations with bone marrow adiposity in subjects chronic and acute SCI, suggesting a connection between mir-148a-3p and the dysregulation of bone remodeling. As miR-148a-3p has been confirmed to participate in osteogenic and adipogenic changes *in vitro*, we propose that the increase in miR-148a-3p during the acute phase of SCI mediates subsequent bone loss in chronic SCI through promotion of adipogenesis of mesenchymal stem cells, as well as increasing bone resorption. Although in our analysis, we have not proven causality, here we present a possible schematic for this mechanism (**Figure 3**). MiR-148a-3p may be a future therapeutic target for the prevention and treatment of SCI induced osteoporosis.

There are limitations of this study to consider. It is a relatively small sample and due to the study design, we were not able to validate miR-148-3p levels in an independent sample. Also, this study was not designed to assess the regulatory roles of miR-148-3p. More research is needed, particularly longitudinal studies to elucidate the mechanisms and magnitude of this effect.

Our findings suggest that miR-148a-3p is a mediator of osteoporosis following spinal cord injury. While more studies are needed to demonstrate causality, miR-148-3p has the potential to be used as a biomarker in identifying people with acute SCI who may go on to develop osteoporosis or as a therapeutic target to prevent this and other bone metabolic SCI sequalae.

## Data availability statement

The original contributions presented in the study are publicly available. This data can be found at Harvard Dataverse: https://doi.org/10.7910/DVN/RGVNRL[accession number: RGVNRL].

## Ethics statement

The studies involving human participants were reviewed and approved by HealthOne IRB Denver CO. The patients/participants provided their written informed consent to participate in this study.

## Author contributions

SL, LM, and RB wrote the manuscript with input from all authors. KT and NM analyzed CT scans and calculated MAT. NN performed the statistical analyses. RB and LM conceived the study and provided direction, planning, and editorial guidance. All authors contributed to the article and approved the submitted version.

## Funding

This study received support from the Department of Defense (Q81XWH-15-2-0078), National Institutes of Health (R01AR064793) and The National Institute on Disability, Independent Living, and Rehabilitation Research (NIDILRR, 90SI5015-01-00). Additional support was provided by the Rehabilitation Research Experience for Medical Students (RREMS) through the Association of Academic Physiatrists, the Foundation for PM&R, and the Craig H. Neilsen Foundation.

## Conflict of interest

The authors declare that the research was conducted in the absence of any commercial or financial relationships that could be construed as a potential conflict of interest.

## Publisher’s note

All claims expressed in this article are solely those of the authors and do not necessarily represent those of their affiliated organizations, or those of the publisher, the editors and the reviewers. Any product that may be evaluated in this article, or claim that may be made by its manufacturer, is not guaranteed or endorsed by the publisher.
